# The Hospitalisation of the Asylum

**Published:** 1916-02-26

**Authors:** 


### THE HOSPITALISATION OF THE ASYLUM.

The employment of female nurses on the male side has not stopped at this stage of evolution in Scotland. Their introduction was in very large part due to the desire to make use in asylums of the high standard of skill in nursing possessed by those who had been trained in our general hospitals. Three-quarters of a century ago, in Dr. Hitch's time, "Sarah Gamp" was in the flesh ("Martin Chuzzlewit," 1843), and there cannot have been much inducement to employ her, or others like her, on the male side for the sake of their skill in nursing. Dr. Hitch's first female nurse attended to refractory patients! If this highly skilled form of nursing be desired, then women must be employed, for unfortunately men do not receive this training in our general hospitals. Large numbers of trained hospital nurses have thus been appointed during the last twelve years to the Scottish asylums in important positions for the sake of their technical skill and training. Dr. Campbell Clark was the first to appoint a trained hospital nurse (Miss Mary Macfarlane) to the post of matron at the Kirklands Asylum, Bothwell, in 1880. In the following year he commenced the systematic teaching and training of his asylum nurses and attendants, as was the practice in general hospitals. The idea took root, and in 1885 the Scottish division published the "Handbook for Attendants on the Insane," the most enterprising action ever taken by a division of the Association. This handbook, as is well known, has since been adopted by the Medico-Psychological Association, and has led to the granting of the certificate for proficiency in mental nursing and to the registration of certificated mental nurses. The first hospital nurse to work within the wards among insane patients and asylum nurses was appointed by me at the Perth District Asylum in 1896. This was an important step not only on account of its direct influence on ward work, but because it created a supply of hospital nurses who were specially trained for the duties of asylum matronship, which had not previously existed. The demand for these became so great that over three dozen of my own nurses have received such appointments in other institutions. As all hospital nurses are accustomed to attend to male patients in the general hospitals, they think it the most natural thing in the world to continue to take charge of male patients in asylums. They have gradually extended the sphere of their usefulness far beyond the limits of the hospital wards on the male side of which they were first appointed, and have invaded other departments. They have introduced innumerable reforms which have approximated the methods employed in the asylums to those in the hospitals, and amongst these the greater employment of women in the male wards is only one. The trend of events in Scotland has been such that this employment of female nurses in the male wards, when seen in its proper perspective, is found to be only a part of a much greater scheme or ideal that has flowed like a tide over the land—that of the hospitalisation of the asylum. It is fifteen years now since this ideal was organised into a working system at the Stirling District Asylum, and a paper describing the methods employed there, entitled "Hospital Ideals in the Care of the Insane," was published by me in the *Journal of Mental Science* in the year 1902. The details of this system go, however, beyond the scope of the present paper, and include the building of asylum hospitals, the bed treatment of the insane, the study of the physical aspects of mental disease, etc.

Time is not only a great healer, but a great judge, who decides most appeals by very convincing logic, and fifteen years is a liberal period in which to test the merits of a system of asylum management. It was said thirteen years ago by a distinguished member of our specialty that the "nursing of male insane patients by females" was "preposterous," and that to run everything in asylums on hospital lines was "a great fad." It must be hinted in palliation that this authority had not had any experience of the methods which he criticised so freely. This method of nursing is now as distinctive and as firmly established a feature of the Scottish system of care for the insane as the well-known boarding-out system. It is employed in some measure or other in all but two of the important asylums of the country, and in these the superintendents have so far failed to introduce it, not because they are opposed to it on principle, but on account of structural difficulties with regard to supervision, housing, etc. This wonderful unanimity of opinion and practice among Scotsmen, whose national proclivities do not tend to concord, is remarkable testimony in its favour, and points to the manifest practicability and overwhelming merits of the system. The hospitalisation of the asylum is still on the whole an ideal to be aspired to, but it, too, is steadily developing and gaining ground year after year. It will perhaps come as a surprise to many to know that in a fourth of the asylums in Scotland, and among them are included some of the large ones, the matron is head of the nursing staff on the male side, as well as on the female. How many hospital nurses are employed as sisters or assistant matrons it is impossible to say, but it must be large, for twenty-two were lately doing duty in the military hospitals. A system such as this, which has survived the test of fifteen years' experience with enhanced reputation and has become national in scope, must now have some other qualification than "preposterous," some other appellation than "a fad," applied to it by all fair-minded and reasonable men and women. The man who sees no good in it, who thinks its adoption impossible, must believe that the Scottish Board of Control and the majority of the medical superintendents in that country, in other respects with the reputation of being shrewd and level-headed, are labouring under an obsession!

